# Opportunities and Challenges of Liquid Biopsy in Thyroid Cancer

**DOI:** 10.3390/ijms22147707

**Published:** 2021-07-19

**Authors:** Chiara Romano, Federica Martorana, Maria Stella Pennisi, Stefania Stella, Michele Massimino, Elena Tirrò, Silvia Rita Vitale, Sandra Di Gregorio, Adriana Puma, Cristina Tomarchio, Livia Manzella

**Affiliations:** 1 Center of Experimental Oncology and Hematology, A.O.U. Policlinico “G. Rodolico—San Marco”, 95123 Catania, Italy; chiararomano83@gmail.com (C.R.); federica.martorana@phd.unict.it (F.M.); perny76@gmail.com (M.S.P.); stefania.stel@gmail.com (S.S.); michedot@yahoo.it (M.M.); elena.tirro@unipa.it (E.T.); silviarita.vitale@gmail.com (S.R.V.); digregoriosandra@hotmail.com (S.D.G.); adry.p88@hotmail.it (A.P.); cristina.tomarchio@hotmail.it (C.T.); 2 Department of Clinical and Experimental Medicine, University of Catania, 95123 Catania, Italy; 3 Department of Surgical, Oncological and Stomatological Sciences, University of Palermo, 90127 Palermo, Italy

**Keywords:** differentiated thyroid cancer, anaplastic thyroid cancer, medullary thyroid cancer, liquid biopsy, diagnosis, prognosis, therapy

## Abstract

Thyroid cancer is the most common malignancy of the endocrine system, encompassing different entities with distinct histological features and clinical behavior. The diagnostic definition, therapeutic approach, and follow-up of thyroid cancers display some controversial aspects that represent unmet medical needs. Liquid biopsy is a non-invasive approach that detects and analyzes biological samples released from the tumor into the bloodstream. With the use of different technologies, tumor cells, free nucleic acids, and extracellular vesicles can be retrieved in the serum of cancer patients and valuable molecular information can be obtained. Recently, a growing body of evidence is accumulating concerning the use of liquid biopsy in thyroid cancer, as it can be exploited to define a patient’s diagnosis, estimate their prognosis, and monitor tumor recurrence or treatment response. Indeed, liquid biopsy can be a valuable tool to overcome the limits of conventional management of thyroid malignancies. In this review, we summarize currently available data about liquid biopsy in differentiated, poorly differentiated/anaplastic, and medullary thyroid cancer, focusing on circulating tumor cells, circulating free nucleic acids, and extracellular vesicles.

## 1. Introduction

Thyroid cancer (TC) is the most frequent endocrine malignancy, accounting for about 2% of total cancers [1,2]. Worldwide, its incidence has increased three-fold over the past 30 years because of screening intensification and environmental and lifestyle changes [3]. The vast majority of TCs have an epithelial origin and include differentiated (DTC), poorly differentiated (PDTC), and anaplastic (ATC) tumors [1,4]. Differentiated TCs, which usually display an indolent clinical behavior and a favorable prognosis, can be further classified as papillary (PTC) (85–90%), follicular (FTC) (5–10%), and Hurthle cell (HCTC) (3%) carcinomas [5,6]. Poorly differentiated and anaplastic TCs are rarer, but they are characterized by an aggressive course and a poor prognosis [7]. Finally, a small proportion of TCs stem from neuroendocrine C-cells and present medullary histotypes (*B-Raf proto-oncogene* MTCs); up to one quarter (25%) of these tumors are familiar [8].

The biological features of TCs have been extensively investigated. Among DTCs, molecular alterations frequently involve *B-Raf proto-oncogene* (*BRAF*) and *Rat Sarcoma* (*RAS*) oncogene or gene fusions involving *Ret proto-oncogene* (*RET*), while deregulation of TP53 and Wingless/Integrated (Wnt)/β-catenin pathways are mainly implicated in their progression and dedifferentiation [9,10,11,12]. In 98% of inherited and up to 45% of sporadic MTCs, single nucleotide substitutions of *RET* are present [13]. Recently, micro-RNAs (mi-RNAs) also appear to play a role in TC biology [14], and different mi-RNAs signatures correlate with the malignant potential of thyroid nodules as well as with TCs subtypes and their aggressiveness [15,16].

**Figure 1:**
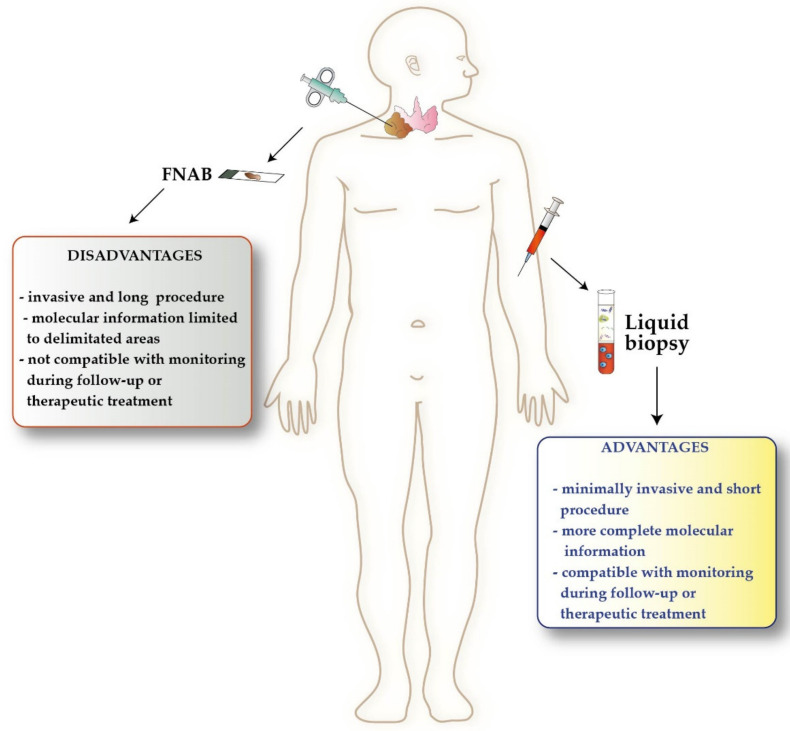
Advantages of liquid biopsy over tissue biopsy in thyroid cancer. FNAB, fine needle aspiration biopsy.

Despite this deep biological knowledge, clinical management of TCs still presents controversies. Although fine needle aspiration biopsy (FNAB) represents the current gold standard for initial diagnosis of TC, it exhibits some limitations due to the high incidence of non-diagnostic results, especially in the case of follicular lesions [17] (Figure 1). In the post-surgical follow-up of DTC, monitoring thyroglobulin (Tg) levels is a routine practice. However, the presence of anti-Tg-antibodies (TgAb) may interfere with Tg measurement, thus hampering its potential utility as a tumor marker [18]. Additionally, while the majority of DTCs can be cured with surgery followed by hormone replacement and radioactive iodine (RAI, also called I-131) [6], the management of advanced, poorly differentiated, and anaplastic TCs is far more challenging [19,20,21]. These tumors can be treated with tyrosine kinase inhibitors (TKIs) [22], such as lenvatinib and sorafenib for DTC, dabrafenib, trametinib, and vemurafenib for BRAF mutated PTC or ATC, cabozantinib and vandetanib for MTC, or selpercaptinib in RET mutated MTCs [10,23,24].

In this scenario, liquid biopsy can be a valuable resource to assist TC management, as it can be exploited to define the correct diagnosis, predict tumor prognosis, monitor disease evolution, and establish pharmacological approaches [25] (Figure 1). Using liquid biopsy, circulating tumor cells (CTCs), circulating free nucleic acids (cf-DNA, cf-RNA and mi-RNA), and tumor-derived extracellular vesicles (EVs) released in the bloodstream can be detected and analyzed [26]. Further, CTCs are shed from the tumor mass and enter circulation, where they can remain unitary, cluster together, or lodge in new tissues to form metastasis [27]. Circulating free-DNA released from cancer cells in 160–180 base pairs fragments (i.e., circulating tumor-DNA (ct-DNA)) can contain information about molecular alterations present in the primary tumor [27,28,29,30]. Circulating free-DNA and circulating mi-RNAs are most stable, thus easier to investigate, compared to cf-RNA [31]. Extracellular vesicles are micro-particles released in the bloodstream from tumor and normal cells containing proteins, DNA, RNA, mi-RNA, lipids, and metabolites [32,33].

In this review, we provide an overview of liquid biopsy applications in different TC histotypes, focusing on its use in the diagnosis, prognostic definition, and treatment of these diseases.

## 2. Liquid Biopsy in Differentiated Thyroid Cancer

### 2.1. Liquid Biopsy in the Diagnosis of Differentiated Thyroid Cancer

**Figure 2:**
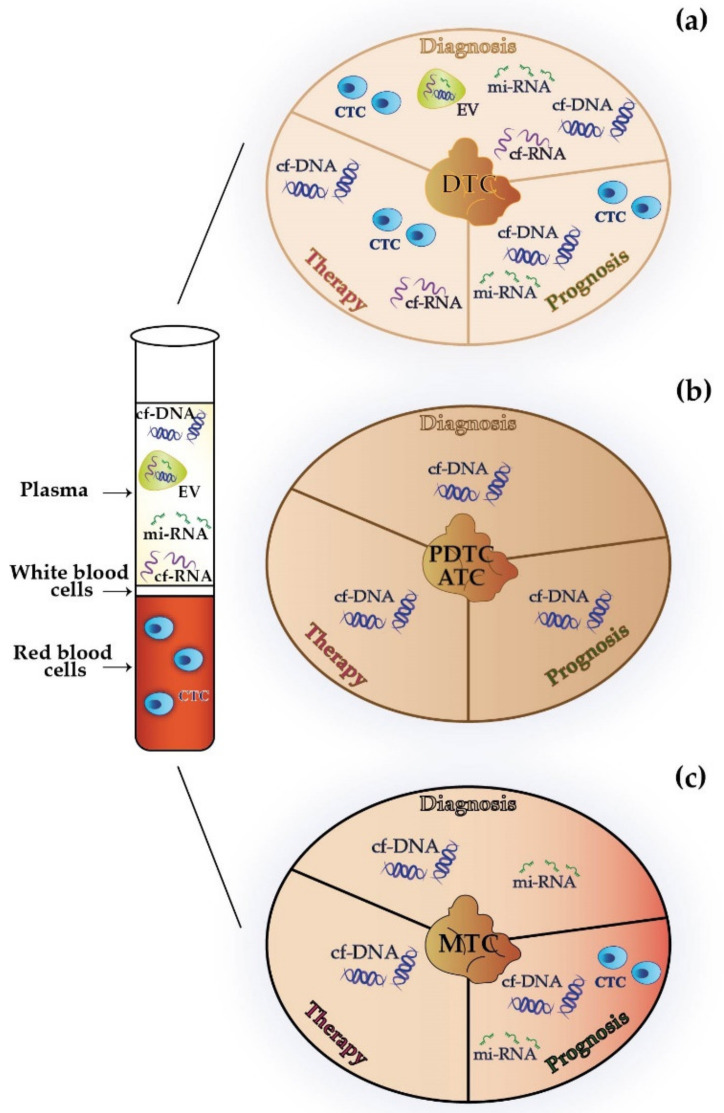
Potential implications of liquid biopsy for the diagnosis, prognostic definition, and therapy of differentiated (**a**), poorly differentiated/anaplastic (**b**), and medullary (**c**) thyroid cancer. cf-DNA, circulating free-DNA; cf-RNA, circulating free-RNA; CTC, circulating tumor cells; DTC, differentiated thyroid cancer; EV, extracellular vesicles; mi-RNA, micro-RNA; MTC, medullary thyroid cancer; PDTC/ATC, poorly differentiated thyroid cancer/anaplastic thyroid cancer.

Circulating tumor cells, cf-DNA, cf-RNA, mi-RNAs, and EVs may represent a source of information in the diagnostic workup of primary or recurrent DTCs (Figure 2a and Table 1).

In the pre-operatory setting, the expression of genes associated with CTCs can help distinguish benign from malignant thyroid nodules with the follicular feature. For example, the expression of the CTC-associated gene *SLC5A5* is lower in liquid biopsies of patients with FTC compared to patients with follicular adenoma (FA), while *LGALS3* expression is higher in PTC than in FTC patients [34]. In the follow-up of surgically removed PTCs, serum levels of circulating epithelial cells (CECs) expressing epithelial cell adhesion molecule (EpCAM) and thyroid-stimulating hormone receptor (TSHR) are significantly higher in patients with disease recurrence and undetectable serum Tg due to TgAb [35].

Evaluating cf-DNA quantity, integrity, mutational profile, and methylation levels can be useful for the diagnostic definition of DTC [60,61]. Indeed, cf-DNA quantity and integrity appear to be higher in patients with a cytological diagnosis of DTC than in unaffected subjects [36,37]. Conversely, mitochondrial cf-DNA (mcf-DNA) is lower in the same subset of patients [37]. Results of the feasibility and significance of *BRAF-V600E* detection in cf-DNA of DTC patients remain controversial [62,63,64,65,66,67]. Although this mutation can be retrieved in cf-DNA from both FTCs and PTCs patients, it is more common in the second histotype [38]. In a retrospective study, measuring the methylation levels of five genes on cf-DNA (*calcitonin*, *E-cadherin*, *tissue inhibitor of metalloproteinase 3*, *death-associated protein kinase,* and *retinoic acid receptor-β2*) displayed 77% accuracy in the differential diagnosis between DTCs and benign nodules [39]. In another study, the decrease of 5-methylcytosine (5mC) and 5-hydroxymethylcytosine (5hmC) levels in cf-DNA predicted the probability of a thyroid cancer diagnosis [40]. More recently, Kathami et al. demonstrated that hypermethylation of *MGMT* promoter in ct-DNA correlates with the presence of PTC [41]. Interestingly, combining the cf-DNA quantity measurement with the detection of *BRAF-V600E*, *SLCA8*, and *SLC26A4* hypermethylation can also assist in the early diagnosis of PTC [42].

Circulating free-RNA has also been investigated as a potential diagnostic marker of DTCs. For instance, *carcinoembryonic antigen* (*CEA*) messenger RNA (mRNA) can be found in the serum of patients with FTC but not in those with benign lesions [43], while blood measurement of *TSHR* mRNA can improve the pre-operative detection of DTC when associated with FNAB [44].

The role of circulating mi-RNAs in the diagnosis of PTC has been extensively investigated and recently reviewed. Comprehensively, a set of mi-RNAs (mi-RNA-375, 34a, 145b, 221, 222, 155, Let-7, and 181b) can be designated as a diagnostic biomarker to distinguish PTCs from benign nodules and identify cancer at an early stage [45]. Additional evidence suggests that plasma-derived mi-RNA-222 and another set of circulating mi-RNAs (let-7a, let-7c, let-7d, and let-7f) can be significantly increased in PTC patients compared to subjects with goiter or healthy controls [46,47]. In a prospective observational study, the serum levels of 754 mi-RNAs were measured in 11 PTC patients before and after surgery. Among these, mi-RNA-146 and mi-RNA-221 were further validated as tumor biomarkers during post-surgical follow-up and showed a significant correlation with disease recurrence, even in patients with low Tg levels [48].

Moreover, specific changes in EV mi-RNA profiles appear to correlate with the development of PTC. Indeed, mi-RNA-31-5p is over-expressed in EVs from PTC patients, while mi-RNA-21 and mi-RNA-181a-5p differentiate FTC from PTC [49]. More recently, the overexpression of four mi-RNAs (Let-7b, Let-7d, Let-7f, and Let-7g) in thyreo-peroxidase (TPO)-positive EVs accurately distinguished FTC from FA [50].

### 2.2. Liquid Biopsy for Prognostic Definition of Differentiated Thyroid Cancer

Liquid biopsy can also be used to predict the clinical course of DTC, by the evaluation of CTCs, cf-DNA, and mi-RNAs (Figure 2a and Table 1).

Using negative enriching immunofluorescence in situ hybridization (NE-iFISH), Qiu et al. showed a potential prognostic implication of CTC count in DTC patients. In their study, the identification of five or more CTCs correlated with the presence of metastasis, while isolating seven or more CTCs predicted poor response to RAI [51]. In another study, the number of CTCs was significantly higher in subjects with a previous DTC compared to healthy controls, and the number of cells isolated was proportional to the tumor stage at diagnosis. Additionally, patients with no evidence of disease recurrence who received RAI >8 years previously had more CTCs compared to those with a shorter treatment-free interval [52].

In a cohort of 57 PTC patients, 24 harbored *BRAF-V600E* in ct-DNA. The presence of this mutation was associated with disease aggressiveness since it correlated with tumor size, multifocal growth, extra-thyroidal gross extension, and pulmonary micro-metastasis [53]. Similarly, an increase in the plasma level of mi-RNA-222 and mi-RNA-146 appears to predict poor outcomes in DTC patients [54].

### 2.3. Liquid Biopsy in the Treatment of Differentiated Thyroid Cancer

Liquid biopsy can also be exploited to monitor response to treatment in patients with DTC. CTC count, ct-DNA, and cf-RNA can potentially outperform serum Tg measurement or radiological imaging as response evaluation methods (Figure 2a and Table 1).

In a pilot study, an early decrease in CEC counts after RAI treatment correlates with disease response in DTC patients [55]. Zheng and colleagues investigated sodium/iodide symporter (NIS) expression in CTCs from DTC patients and found a correlation between a decreased or unchanged number of total NIS+ CTCs and the efficacy of RAI therapy [56].

According to a study by Allin et al., changes in ct-DNA levels can anticipate tumor progression compared to Tg in patients receiving targeted therapies for DTC [57]. Using co-amplification at lower denaturation temperature-PCR (COLD-PCR) in combination with digital droplet polymerase chain reaction (ddPCR), Jensen et al. identified *BRAF-V600E* mutation cf-DNA in 57 PTC patients. According to their results, *BRAF-V600E*-mutated patients display nearly five-fold higher odds of achieving an incomplete response to RAI [53]. Measurement of *BRAF-V600E* mutant ct-DNA can also be informative of the presence of minimal residual disease. In a cohort of *BRAF-V600E* mutated PTC patients, levels of *BRAF-V600E* mutant ct-DNA were higher in the case of disease persistence (0–2.07%) compared to no evidence of disease (0–0.04%). Similarly, ct-DNA copy numbers were higher in patients with metastases (20 copies/mL) than in those without residual disease after thyroidectomy (1 copy/mL) [58].

Additionally, the levels of *BRAF-V600E* cf-RNA appear to decrease after surgery and during systemic treatment with RAI or TKIs in patients with early, recurrent or advanced PTC [59].

## 3. Liquid Biopsy in Poorly Differentiated and Anaplastic Thyroid Cancer

Poorly differentiated and anaplastic thyroid tumors represent rare and aggressive entities and little is known about the role of liquid biopsy in their management.

In some real-world experiences, cf-DNA has been employed in ATC patients to identify actionable mutations (i.e., *BRAF-V600E*) [68,69] and to anticipate disease response or progression before they become radiologically apparent [70] (Figure 2b and Table 2).

Recently, Qin et al. examined the concordance of ATC-related mutations in cf-DNA with those detected in tumor tissue, trying to determine the prognostic significance of cf-DNA mutations. As expected, *TP53*, *BRAF,* and *PIK3CA* were the most frequently mutated genes. In 28 treatment-naïve ATC patients, the concordance rate of detected mutations in *TP53*, *BRAF,* and *PIK3CA* between cf-DNA and matched tissue was 82.1%, 92.9%, and 92.9%, respectively. Moreover, patients with a *PIK3CA* mutation detected on cf-DNA had worse overall survival (OS) [71]. The same group evaluated the employment of ddPCR for the identification of *BRAF-V600E* mutation on cf-DNA in 44 ATC patients, finding a 93% concordance rate with DNA sequencing on tumor tissue. Additionally, dynamic measurement of *BRAF-V600E* levels by ddPCR during treatment was available for 16 patients. Whether the reduction in circulating biomarker levels correlated with tumor shrinkage, their increase was weakly associated with disease progression [72]. However, other research found that an increase in mutations in circulating *NRAS* and *TP53* in a PDTC and an ATC patient anticipated, by several months, radiological disease progression [57].

## 4. Liquid Biopsy in Medullary Thyroid Cancer

Several studies are accumulating about the use of liquid biopsy in MTC patients to detect and analyze CTCs, cf-DNA, and mi-RNAs (Figure 2c and Table 3).

Circulating tumor cells showed a prognostic and predictive role in this subset of patients. In a recent report, calcitonin positive (Ctn+) CTCs were identified in the serum of 15 patients with surgically removed MTC, up to 12 years after initial diagnosis. Of note, high CTC levels were found in the serum of three MTC patients with low blood Ctn. According to the authors, this group of patients may have a poorer prognosis; further validation of these results in larger cohorts are needed [76]. Older reports suggest that cytokeratin 20 (CK20) and gastrine-releasing peptide (GRP) expression can also be used to identify MTC-derived CTCs with good sensitivity and specificity [82]. According to other evidence, the detection and enumeration of CTCs with the approved EpCAM-based CellSerch technology can predict OS and mortality risk in patients with advanced MTC [77,78]. The identification of five or more CTCs in patients with metastatic MTC appears to predict shorter survival [79].

The role of cf-DNA as a marker of tumor diagnosis, prognosis and response to treatment has been reported in MTC. For example, Ctn serum levels and the presence of *RET* mutation inversely correlated with cf-DNA amount in 58 MTC patients. Hence, cf-DNA may serve as a diagnostic marker of MTC when conventional parameters, such as Ctn and RET, are negative [42]. Similarly, the detection of *RET-M918T* in cf-DNA appear to predict MTC outcomes more accurately than Ctn doubling time, strongly correlating with worse OS [80]. In a study by Allin and al., *RET* and *BRAF* mutations were identified in a cohort of 15 MTC, with a detection rate in cf-DNA of 79%, higher than that found in PTC and FTC patients. Mutations were more frequently detected in MTC patients with metastasis, high tumor burden, and progressive disease, thus predicting an unfavorable prognosis [57]. More recently, *RET* mutations were identified in the cf-DNA of MTC patients developing disease progression after an initial response to selpercaptinib. In this study, *RET-V804M* mutation, detected in two patients before treatment initiation, decreased during therapy and reappeared together with *RET-G810* mutations at the start of disease progression. As the detection of these mutations has been linked with selpercaptinib resistance, ct-DNA monitoring may facilitate the early identification of unresponsive patients who need alternative therapies [81].

Circulating mi-RNAs can be easily obtained from MTC patients, and their levels correlate with clinical-pathological features and disease prognosis [83]. Zhang et al. analyzed the expression of serum mi-RNA in 15 patients with aggressive MTC. Circulating mi-RNA-222-3p and mi-RNA-17-5p were significantly upregulated in MTC patients and discriminated between subjects with MTC and those with benign or healthy nodules [73]. Romeo et al. identified 51 mi-RNAs differentially expressed in a cohort of locally advanced and metastatic MTC patients. Among them, mi-RNA-375 levels were significantly higher in patients with active disease than in those unaffected or cured. Of note, elevated levels of mi-RNA-375 correlated with distant metastasis and reduced OS, but not with disease response to vandetanib [74]. Another study evaluated plasma levels of mi-RNA-144 and mi-RNA-34a in 25 RET-mutant and 25 RET-wild type MTC patients and compared them to healthy controls. According to their results, blood levels of mi-RNA-144 and mi-RNA-34a were higher in cancer patients, especially if RET mutant, than in controls. However, these data were not significantly associated with MTC prognosis [75].

## 5. Conclusions

In this review, we discussed the potential applications of liquid biopsy in TC. As the incidence of thyroid carcinomas is increasing over time, more effective tools for the management of these tumors are needed. In this context, liquid biopsy is a promising alternative during the diagnostic workup, prognostic definition, therapeutic choices, and follow-up of TC patients.

Liquid biopsy offers many advantages compared to traditional tissue biopsy, such as less invasiveness, few side-effects, repeatability, and representativeness of tumor heterogeneity. Moreover, the applications of liquid biopsy are continually broadening [84], with the development of new techniques such as CellSearch^®^ and DEPArray^®^ for CTC detection and characterization, or automation of ct-DNA isolation [30]. Given the rapid evolution and improvement in this field, other applications may emerge in the future for the management of different TC subtypes. To pursue personalized medicine, *BRAF* mutations in PTC or *RET* alterations in MTC can be routinely screened through liquid biopsy to assess sensitivity towards targeted agents and to monitor the onset of resistance.

However, the use of liquid biopsy in TC still poses several challenges, mainly depending on the sensitivity and specificity of the different methods, and tumor subtypes and stages Additionally, liquid biopsy often displays standardization, reproducibility, and validity issues [85]. Other technical problems may rely on the quantity of circulating material retrieved, especially in the context of early-stage disease and in terms of CTCs and ct-RNA. This latter aspect may represent a major limitation of liquid biopsy in TCs harboring pathogenic rearrangements, such as those involving *RET* or the *neurotrophic receptor tyrosine kinase* (*NTRK*).

In conclusion, although the clinical utility of liquid biopsy in TC is progressively consolidating over time, efforts are needed to incorporate this technology into clinical practice, as already happens in many other tumor types, e.g., lung cancer [86]. Even though it is unlikely that liquid biopsy will completely substitute tissue biopsy, soon the two techniques may be complementary. In this context, international consortia such as the European Liquid Biopsy Society and the US-based BloodPAC are working to move liquid biopsy from the bench to the bedside.

## Figures and Tables

**Table 1:** Potential implications of liquid biopsy for the diagnosis, prognostic definition, and therapy of differentiated thyroid cancer.

Clinical Application	Sample Type	Object	Modification	References
Diagnosis	CTC	Expression of the CTC-associated gene *SLC5A5*	Lower in FTC than FA	[34]
		Expression of the CTC-associated gene *LGALS3*	Higher in PTC than FTC	[34]
		Serum level of CEC	Higher in recurrent PTC	[35]
	cf-DNA	cf-DNA quantity and integrity	Higher in DTC	[36,37]
		mcf-DNA	Lower in DTC	[37]
		*BRAF-V600E* detection	More common in PTC than FTC	[38]
		Metilation level of 5 genes on cf-DNA	Higher in DTC than benign nodules	[39]
		5mC and 5hmC levels in cf-DNA	Decreased in TC	[40]
		Hypermethylation of *MGMT* promoter in ct-DNA	Higher in PTC	[41]
		cf-DNA quantity, *BRAF-V600E* detection, *SLCA8* and *SLC26A4* hypermethylation	Coexist in PTC	[42]
	cf-RNA	Serum level of *CEA* mRNA	Found in FTC but not in FA	[43]
		Serum level of *TSHR* mRNA	Higher in DTC	[44]
	mi-RNA	mi-RNA-375, 34a, 145b, 221, 222, 155, Let-7, 181b	Higher in PTC than benign nodules	[45]
		mi-RNA-222	Higher in PTC	[46]
		let-7a, let-7c, let-7d, let-7f	Higher in PTC	[47]
		mi-RNA-146 and mi-RNA-221	Higher in recurrent PTC	[48]
	EV	mi-RNA-31–5p	Higher in PTC	[49]
		mi-RNA-21 and mi-RNA-181a-5p	Differentiated FTC from PTC	[49]
		Let-7b, Let-7d, Let-7f and Let-7g	Higher in FTC than FA	[50]
Prognosis	CTC	CTC count	Higher in metastatic DTC or poor responder to RAI	[51]
		CTC number	Higher in advanced DTC stage	[52]
	cf-DNA	*BRAF-V600E* detection	Associated with disease aggressiveness in PTC	[53]
	mi-RNA	mi-RNA-222 and mi-RNA-146	Higher in DTC with poor outcome	[54]
Therapy	CTC	CEC count	Decreased in DTC responding to RAI therapy	[55]
		CTCs expressing NIS	Decreased in DTC responding to RAI therapy	[56]
	cf-DNA	ct-DNA levels	Higher in poor responder or unresponsive DTC	[57]
		*BRAF-V600E* detection	Associated to incomplete response to RAI in PTC	[53]
		*BRAF-V600E* detection	Higher in PTC with minimal residual disease	[58]
		ct-DNA copy number	Higher in PTC with residual disease after surgery	[58]
	cf-RNA	Level of *BRAF-V600E* cf-RNA	Decrease in PTC during RAI or TKIs treatment	[59]

CEA, carcinoembryonic antigen; CEC, circulating epithelial cells; cf-DNA, circulating free-DNA; cf-RNA, circulating free-RNA; CTC, circulating tumor cell; ct-DNA, circulating tumor-DNA; DTC, differentiated thyroid cancer; EV, extracellular vesicles; FA, follicular adenoma; FTC, follicular thyroid cancer; mi-RNA, micro-RNA; PTC, papillary thyroid cancer; RAI, radioactive iodine; TC, thyroid cancer; TKI, tyrosine kinase inhibitors; TSHR, thyroid stimulating hormone receptor.

**Table 2:** Potential implications of liquid biopsy for the diagnosis, prognostic definition, and therapy of poorly differentiated/anaplastic thyroid cancer.

Clinical Application	Sample Type	Object	Modification	References
Diagnosis	cf-DNA	*TP53*, *BRAF* and *PIK3CA* mutations	Concordance with mutations retrieved in tumor tissue	[71]
		*BRAF-V600E* detection		[72]
Prognosis	cf-DNA	*PIK3CA* mutation	Worse OS in ATC	[71]
		*BRAF-V600E* detection	Increased in ATC progression	[72]
		*NRAS* and *TP53* mutations	Higher in progressive PDTC and ATC	[57]
Therapy	cf-DNA	*BRAF-V600E* detection	Indication for TKI treatment	[68,69]

ATC, anaplastic thyroid cancer; cf-DNA, circulating free-DNA; OS, overall survival; PDTC, poorly differentiated thyroid cancer; TKI, tyrosine kinase inhibitor.

**Table 3:** Potential implications of liquid biopsy for the diagnosis, prognostic definition, and therapy of medullary thyroid cancer.

Clinical Application	Sample Type	Object	Modification	References
Diagnosis	cf-DNA	cf-DNA amount	Higher in MTC	[42]
	mi-RNA	mi-RNA-222-3p and mi-RNA-17-5p	Higher in MTC	[73]
		mi-RNA-375	Higher in active disease	[74]
		mi-RNA-144 and mi-RNA-34a	Higher in RET-mutant cancer	[75]
Prognosis	CTC	Ctn+ CTC level	Higher in MTC with worse prognosis	[76]
		CTC detection	Concordance with OS and mortality risk	[77,78]
		CTC detection	Higher in metastatic cancer	[79]
	cf-DNA	*RET-M918T* detection	Correlation with worse OS	[80]
		*RET* and *BRAF* mutations	Concordance with unfavorable prognosis	[57]
	mi-RNA	mi-RNA-375	Higher in metastatic cancer and reduced OS	[74]
Therapy	cf-DNA	*RET* mutations	Correlation with selpercaptinib resistance	[81]

cf-DNA, circulating free-DNA; CTC, circulating tumor cell; ctn+, calcitonin positive; mi-RNA, micro-RNA; MTC, medullary thyroid cancer; OS, overall survival.
